# Etching Rate Analysis Model Based on Quartz Bond Angle Characteristics

**DOI:** 10.3390/mi15060768

**Published:** 2024-06-08

**Authors:** Xinjia Zhao, Chengbao Lv, Shuanqiang Song, Meng Zhao, Jing Ji

**Affiliations:** 1 Research Center for Applied Mechanics, School of Electro-Mechanical Engineering, Xidian University, No.2 South TaibaiRoad, Xi’an 710071, China; 21041110437@stu.xidian.edu.cn (X.Z.); jingji@xidian.edu.cn (J.J.); 2 Shaanxi Key Laboratory of Space Extreme Detection, Xi’an 710071, China

**Keywords:** quartz, etching rate, bond angle characteristics, MEMS

## Abstract

This paper proposes a method for classifying crystal planes based on the bond angle characteristics of quartz unit cells and constructs an etch rate model for quartz crystal planes at both macro and micro scales. By omitting oxygen atoms from the quartz cell structure, a method based on bond angle characteristics was established to partition the atomic arrangement of the crystal surface. This approach was used to analyze the etching processes of typical quartz crystal planes (R, r, m, and (0001)), approximating the etching process of crystals as a cyclic removal of certain bond angle characteristics on the crystal planes. This led to the development of an etch rate model based on micro-geometric parameters of crystal planes. Additionally, using the proposed bond angle classification method, the common characteristics of atomic configurations on the crystal plane surfaces within the X_cut type were extracted and classified into seven regions, further expanding and applying the etch rate model. The computational results of this model showed good agreement with experimental data, indicating the rationality and feasibility of the proposed method. These also provide a theoretical basis for understanding the microstructural changes during quartz-based MEMS etching processes.

## 1. Introduction

Wet etching technology, due to its low cost and suitability for mass production, is commonly employed in the fabrication of MEMS devices [1,2]. However, the complex anisotropy and sidewall defects in MEMS wet etching fabrication may lead to undesired outcomes in the etched structures [3]. Therefore, precise etching and shaping of crystalline materials are crucial for fabrication, and a comprehensive understanding of the etching characteristics of materials is an effective approach to improve the design and fabrication of MEMS devices [4,5,6]. Quartz crystals, being a unique material, demonstrate stable properties over a broad temperature range, along with distinctive piezoelectric characteristics. This facilitates integrating drive and sensing electrodes within a single resonator, thus minimizing alignment errors common in high-end sensors [7]. Consequently, quartz is widely used in manufacturing MEMS devices [8,9]. In wet etching, quartz crystals are typically etched using saturated ammonium fluoride (NH_4_HF_2_) or buffered oxide etchant (BOE), the latter being a blend of NH_4_F:HF. However, owing to their trigonal crystal system and dual-atomic crystal structure, quartz crystals exhibit complex anisotropic etching properties [10,11]. Overall, characterizing the anisotropy (or orientation dependence) of etch rates for various crystal planes is crucial to understanding the propagation of the etch front in high-precision micro-nano structure etching processes [12]. Therefore, analyzing the anisotropy of quartz crystal etch rates at the microscopic atomic level to reveal the intrinsic causes of this anisotropy will benefit the improvement of MEMS wet etching processes and the development of corresponding simulation models.

At the microscopic atomic level, variations in etching rates among crystal planes are the primary cause of anisotropic etching in crystal microstructures, providing a significant basis for analyzing etching variations and optimizing processes [13,14,15]. Furthermore, relevant studies indicate that the arrangement of surface atoms significantly impacts the etching rate of crystal planes [16,17]. Therefore, investigating the differences in etching rate variations between crystal planes from a microscopic atomic level is essential for understanding the anisotropy of quartz crystals. Zubel categorized surface atoms in single-crystal silicon based on bond type and calculated the time needed to break bonds during etching. This allowed the determination of the etch rate of silicon crystal planes by calculating the ratio of the vertical distance between adjacent surface atom layers to the time required for bond breaking [18]. Similarly, Zhou et al. put forth the idea that the crystal etching process involves sequential removal of surface atoms, with the removal time of several atomic layers equating to the advancement time of the crystal surface (or a certain etching depth) [19]. This approach, akin to Zubel’s, resolves the etching rate of crystal planes by considering the time required for chemical bond fracture or the removal of individual atoms during etching. Gosálvez and team proposed a step-terrace pattern for the periodic arrangement of crystal surface atoms, where the most active atoms at step edges are first removed during etching. Then, the terrace atoms adjacent to the steps become step-edge atoms, a process termed step-flow [20,21]. The simplicity of the diamond arrangement of monocrystalline silicon cells enables the direct utilization of the crystallographic structure in defining the neighborhood and atomic type of different surface positions, thereby establishing the relationship model between etching rate and crystal surface atomic structure. In recent years, Wu et al. simplified the oxygen atoms in the sapphire cell and applied the step-flow mechanism to the simplified sapphire atomic model, deriving theoretical etching rate data under different experimental conditions [22]. Following this, Guo et al. employed the step-flow model to describe the anisotropic wet chemical etching of gallium nitride and derived a mathematical expression for the etching rate of surface orientations in the <0001> crystal region [23].

**Figure 1:**
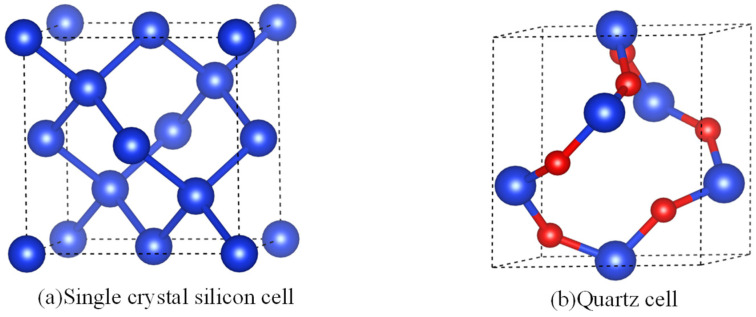
Schematic diagram of monocrystalline silicon and quartz cell models.

Although the methods mentioned above have been applied preliminarily to silicon, simplified sapphire crystal models, and gallium nitride materials, quartz crystals, with their spiral crystal structure of diatomic units, exhibit a more irregular arrangement of surface atoms compared to other crystal models. Figure 1a depicts the single crystal silicon unit cell model, which shares an identical structure with diamond, consisting of two interpenetrating face-centered cubic primitive lattices [24]. Nevertheless, the presence of oxygen atoms within the quartz crystal lattice, along with the double molecular chain structure (Si-O-Si-O), significantly increases the complexity of its crystal surface atomic arrangement compared to single crystal silicon, as depicted in Figure 1b. Employing previous methods, such as distinguishing surface atom types based on chemical bonds or the number of neighboring atoms of target atoms, for irregular crystal planes may lead to the identification of up to a dozen distinct atom types. This proliferation of atom types requires the determination of additional unknown parameters for atomic detachment rates, thereby complicating the solving process. Moreover, the irregular and intricate surface atom arrangement on quartz crystal planes poses challenges in identifying step structures or may result in their complete absence, impeding the extension of the etch rate model based on step-flow theory to quartz crystal materials. Consequently, the complex structural nature of quartz crystals has led to a scarcity of literature on the etch rate models of quartz crystals in recent years. Hence, pursuing a simpler method to classify the arrangement of surface atoms on quartz crystal planes and establishing a relationship model between surface atom structure arrangements and macroscopic etch rates is a more effective approach. Zhang et al. successfully reduced simulation program memory usage and achieved a high level of agreement between simulation and experimental results by simplifying the Si-O-Si bonds to Si-Si bonds [25]. In essence, this simplified model demonstrates feasibility. This approach, which focuses on preserving the original crystal structure of quartz while simplifying the retention of atomic neighbors, makes it possible to establish a relationship between quartz crystal surface atomic types and etch rate models. Building on this concept, this study adopts a simplified quartz unit cell model, focusing on the bond angle characteristics on various crystal planes. The paper proposes that the bond angle features of surface atoms on different crystal planes and micro-geometric parameter information are fundamental reasons for the anisotropy of etch rates, thus facilitating the division and classification of different crystal planes and the construction of etch rate models at macro and micro scales.

This paper’s structure is outlined as follows. Section 2 is subdivided into three main parts: (i) introduction of a simplified model of quartz crystals and a method for atomic group division based on bond angle characteristics of typical crystal planes; (ii) exploration of the etching mechanism of crystals from a micro-atomic level, utilizing crystal plane geometric parameters to elucidate crystal plane anisotropy; and (iii) division of crystal planes within the X_cut orientation based on bond angle characteristics and determination of basic crystal planes, thereby further expanding the etch rate model. Section 3 presents the experimental results and discussions of this study, while the conclusion is provided in Section 4. 

## 2. Atomic Division of Crystal Plane and Analysis of Etching Rate Model

### 2.1. Method for Atomic Division of Crystal Surface

**Figure 2:**
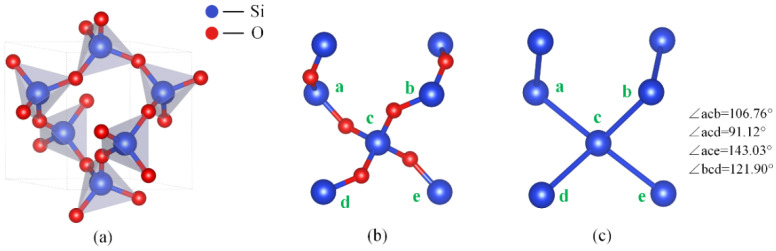
Schematic diagram of quartz crystal atoms. (**a**) Single quartz cell structure; (**b**) traditional quartz crystal structure; (**c**) simplified quartz crystal structure.

Figure 2a depicts the structure of a single quartz unit cell, wherein each Si atom is bonded to four oxygen atoms, forming a tetrahedron. Further analysis revealed that each oxygen atom composing the quartz unit cell is consistently positioned between two Si atoms, arranged predominantly in two fixed molecular chains (-Si-O-Si-O-) (as depicted in Figure 2b). This specific arrangement results in varied etching rates along crystallographic directions [26]. To visualize the etching process of quartz crystals at the atomic layer level, some researchers have simplified the quartz unit cell structure by removing all oxygen atoms and connecting Si atoms via chemical bonds to create a simplified version of the quartz unit cell (as shown in Figure 2c). Subsequently, they have employed Monte Carlo methods to simulate the etching morphology of Z_cut quartz substrates [25]. This simplified model, by disregarding the oxygen atoms within the quartz unit cell, greatly simplifies the arrangement of quartz crystal atomic structures while retaining the overall structural type of the quartz unit cell. Specifically, it removes oxygen atoms, but the spatial positions of silicon atoms and the crystal cell structure remain unchanged. Given its successful application in etching simulation methods, this model provides a feasible crystal cell simplification scheme and reduces the difficulty of analyzing different crystal planes at the microscopic atomic level. By constructing this simplified unit cell model, this study identified that the bond angles between Si-Si bonds predominantly manifest in four forms: 91.12°, 106.76°, 123.9°, and 143°, as depicted in Figure 2c. Based on this observation, this study hypothesized that bond angles formed by surface atoms on crystal faces correlate with these angles, and various crystal plane characteristics emerge from the combinations of these bond angles.

**Figure 3:**
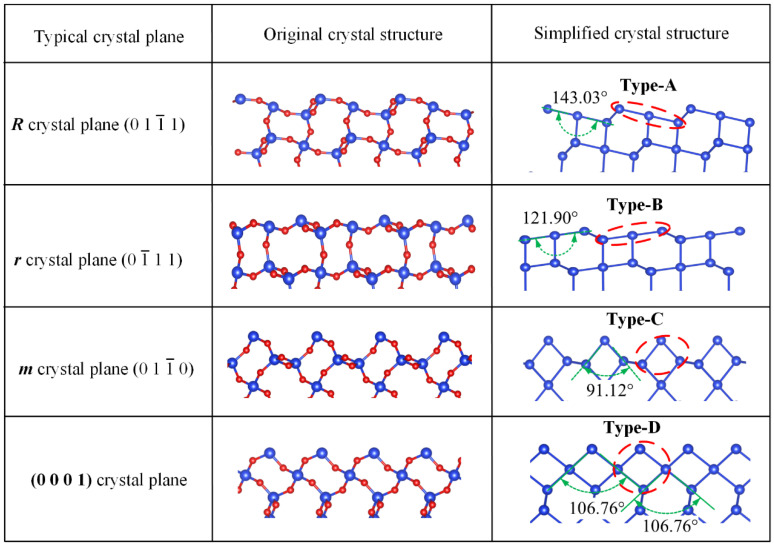
Typical crystal plane and simplified model.

In the trigonal crystal system, quartz crystals can be macroscopically approximated as consisting of m, R, and r crystal planes, demonstrating triadic symmetry. The microscale atomic arrangements of the R and r planes exhibit a remarkable similarity, despite their distinct etching rates. Based on a simplified model of quartz, this study hypothesized a correlation between atomic arrangements on various crystal planes and the size characteristics of surface atomic bond angles. Therefore, this paper aimed to validate this hypothesis by analyzing the surface atomic arrangements of typical crystal planes of quartz, including the commonly used (0001) plane. Figure 3 depicts the analysis of several typical crystal planes. Measurement of bond angle characteristics among atoms on these crystal planes revealed consistent representation through a specific bond angle feature, forming a cyclic arrangement within the entire crystal plane structure. For example, the surface atoms of the R plane mainly consist of atoms with a bond angle of 143.03°, and the internal atomic arrangement is repeated with a nearly identical structure. To simplify analysis, we denoted this bond angle characteristic as atomic group Type-A, aiming to streamline surface atom analysis and explore the feasibility of distinguishing crystal planes using this method. Similarly, we analyzed the surface atom bond angles of the r, m, and (0001) crystal planes using the same method, categorizing them as atomic groups Type-B, Type-C, and Type-D based on respective bond angles of 121.90°, 91.12°, and 106.76°. This bond angle-based atomic group division effectively characterizes internal atomic arrangements within crystal planes. Consequently, by analyzing these typical crystal planes, it was apparent that this approach enables the differentiation of various crystal planes, opening avenues for exploring relationship models between them and etch rates.

### 2.2. Analysis of Etching Mechanism

The factors influencing the crystal etching process can generally be considered from two perspectives: (1) geometric factors, mainly related to crystal structure and surface atomic configuration; (2) chemical factors, depending largely on the type and composition of the etching solution, as well as additional process parameters. Chemical factors are considered as experimental variables, characteristic of etching conditions, and various process parameters can yield different experimental results [27,28]. At the microscopic level, the anisotropy and the mechanism of atomic removal during crystal etching are undoubtedly related to the atoms on different crystal planes. Based on Section 2.1, which classifies the surface atoms of crystal planes by bond angle characteristics (i.e., atomic grouping mode), we attempted to analyze the differences between various crystal planes from a geometric perspective, thereby assessing the feasibility of constructing the feasibility of etch rate equations for crystal planes.

**Figure 4:**
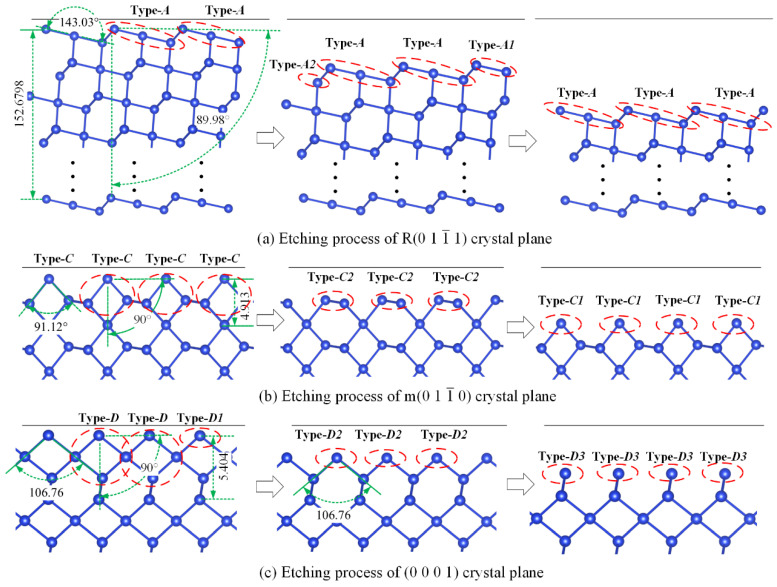
Analysis of etching process of different crystal planes.

Figure 4a depicts the surface atomic structure of the R$\left(0\;1\;\bar{1}\;1\right)$ crystal plane. Analysis of the bond angles among its surface atoms revealed that this crystal plane can be represented by atomic group Type-A. During the etching process, if the morphology of the etched surface is disregarded, the initial surface atoms typically come into contact with the etchant first and react, leading to the successive removal of surface atoms layer by layer and gradual inward etching into the crystal. Consequently, it can be considered that the outermost surface atoms are first stripped off during the etching process. As depicted in Figure 4a, when a layer of surface atoms was stripped away, the exposed surface atomic group configuration closely resembled the initial crystal plane atomic group configuration (the surface atomic configuration can be considered as laterally extending infinitely, with the combination of sub-atomic groups Type-A1 and Type-A2 forming atomic cluster Type-A). This indicates that the etching process of the crystal plane can be viewed as a dynamic repetitive process; that is, reproducing the surface atomic configuration forms an etching cycle, with corresponding distances and angle information of 152.67 Å and 89.98°, respectively. Given the similar arrangement of surface atomic groups between the r$\left(0\;\bar{1}\;1\;1\right)$ plane and the R plane, albeit with differing bond angles, the analytical approach aligns with Figure 4, eliminating the necessity for further graphical depiction. Similar methods were used to analyze the etching processes of the m$\left(0\;1\;\bar{1}\;0\right)$ and Z_cut crystal planes. As depicted in Figure 4b,c, the surface atoms of the m plane can be represented by atomic group Type-C with a bond angle of 91.76°. Stripping one Type-C (comprising two layers of surface atoms, i.e., sub-atomic clusters Type-C1 and Type-C2) from this crystal plane forms a dynamic cycle, with the etching depth to reproduce the surface atomic configuration being 4.913 Å. The etching process of the (0001) crystal plane differs from the other three types. It can be represented by a rhombus structure Type-D with a surface bond angle of 106.76°, requiring the removal of three layers of surface atoms (sub-atomic groups Type-D1, Type-D2, and Type-D3 that form atomic group Type-D) to complete one cycle, with an etch depth distance of 5.404 Å, as shown in Figure 4c. Further analysis revealed that the atomic group configurations formed by sub-atomic groups Type-D1 and Type-D2, and Type-D2 and Type-D3, all had a bond angle of 106.76° (as shown in Figure 4c). Based on the analysis of these typical crystal planes’ etching processes, it is evident that if a crystal plane can be characterized by atomic groups, then the structural etching process of the crystal can be considered a dynamic repetitive process. This means that the etching process can be approximately viewed as continuous cyclic etching of a specific type of atomic group until the stripped crystal plane reveals an atomic arrangement consistent with the initial crystal plane atomic group, thereby determining the etching depth. The periodic arrangement exhibited by the crystal at the microscopic atomic level ensures that the initial crystal plane and the exposed surface atomic clusters after stripping maintain a high degree of structural similarity at certain points. Consequently, at the microscopic atomic level, the detachment rate of each layer of types of atomic groups can be approximately equal after detaching *N* layers of specific types of atomic groups to form an etching cycle and determine etching depth. The crystal’s structural characteristics determine that the spacing between each layer of the type of atomic groups remains constant, thereby establishing a relationship between microscopic atomic planes and macroscopic etching rate. One can assume that the macroscopic etching rate is uniformly distributed among each layer of detached types of atomic groups. According to the step-flow mechanism, the state variable of the atom at the beginning of etching is 1, and the time to remove this atom from the crystal plane is represented by Δ*t* = 1/*R* (where *R* is the detachment rate of the target atom) [20,21]. Thus, considering the atomic groups on the plane as a whole, the time for the removal of the atomic groups can be similarly approximated as Δ*t* = 1/*R_type_*. Therefore, the following derivation can be concluded as:

(1) $$\frac{Deep}{{1/R}_{type}}=\frac{R_{rate}}{N}$$

where *R_rate_* denotes the macroscopic etching rate data of the crystal plane, and *Deep* represents the etching depth, defined as the distance that reproduces the surface atomic positions and their corresponding positions on the initial surface atoms, with the angle between them approximated to 90°. *N* represents the number of layers of atoms removed from the crystal plane to replicate the initial surface atomic structure. *R_type_* represents the etching rate of removing atomic groups with a certain type of configuration. It is essential to note that unlike Type-A and B configurations, Type-C and Type-D configurations typically comprise two or three subatomic groups. The number of stripped layers *N* cannot be directly obtained but can be indirectly determined based on the etching depth *Deep* and the distance of a single Type-C or Type-D configuration.

### 2.3. Partition of X_cut Crystal Plane and Selection of Basic Crystal Plane

**Figure 5:**
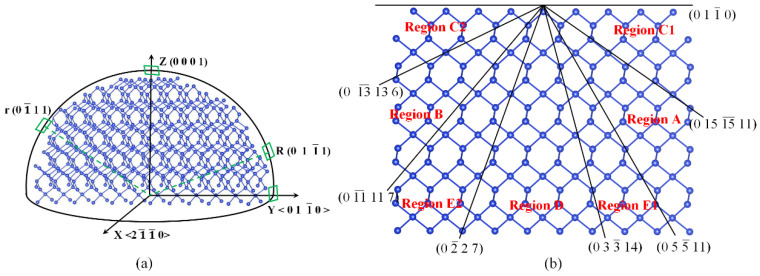
Identification and division of X_cut crystal plane. (**a**) The distribution of crystal planes in the $\langle 0\;1\;\bar{1}\;0\rangle$ crystallographic zones on the simplified quartz model, and (**b**) simplified atomic structure of quartz observed from $\langle 0\;1\;\bar{1}\;0\rangle$ and $\langle 2\;\bar{1}\;\bar{1}\;0\rangle$ crystallographic orientations, respectively.

**Figure 6:**
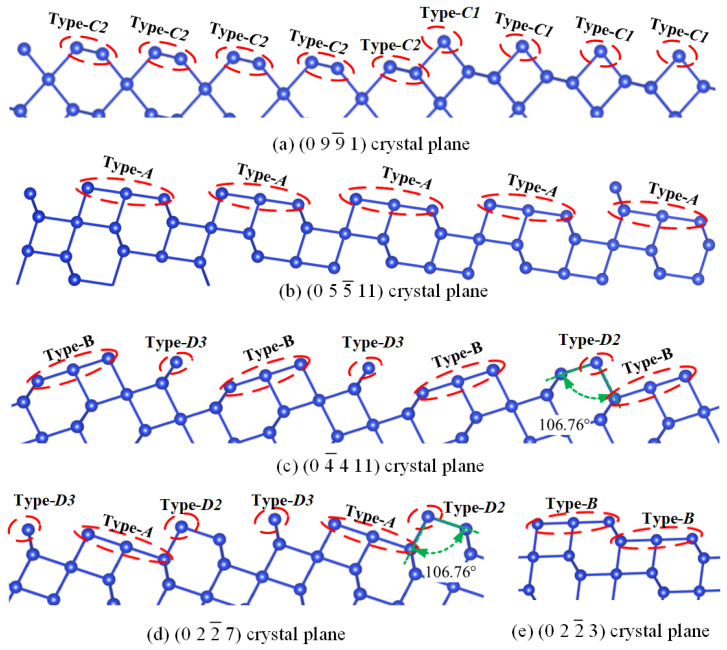
Surface group arrangement of some crystal planes.

Builds upon the analysis conducted in Section 2.1 and Section 2.2 to examine the common characteristics of various typical crystal planes (R, r, m, and (0001)) and their neighboring regions by employing a method of dividing atomic groups’ structure. It then extends this method to other crystal planes or regions to construct intrinsic connections between different crystal planes. To validate this speculative hypothesis, the crystal planes within the quartz X_cut type were analyzed. This cut type was primarily chosen because it is relatively easier to obtain and, compared to the Z_cut type, has a wider range of crystal plane etching rate data and includes typical quartz crystal planes, as shown in Figure 5a. By dividing the surface atoms of the crystal planes within the X_cut type into atomic groups (Figure 5b), this study found that all crystal planes within this region can be represented by one or two atomic groups or sub-atomic group structures from the typical crystal planes in Figure 3, either individually or in combination. For example, the $\left(0\;9\;\bar{9}\;1\right)$ crystal plane in Figure 6a can be composed of one atomic group, Type-C, which includes sub-atomic groups Type-C1 and Type-C2. Similarly, the $\left(0\;2\;\bar{2}\;7\right)$ crystal plane can be composed of a mixture of Type-A and Type-D atomic groups or sub-atomic groups. Therefore, this method of partitioning crystal planes based on bond angle characteristics demonstrates its feasibility. Furthermore, by analyzing common features in surface atomic group configurations of crystal planes within the X_cut orientation, these crystal planes were classified into seven regions. The surface atomic arrangement of each region’s crystal planes can be represented by the same type of atomic group or sub-atomic group configuration, either individually or in combination. Hence, the atomic group configurations in each region’s crystal planes are essentially consistent. For example, the surface atomic arrangements of all crystal planes in Regions A and B can be represented by atomic group configurations Type-A and Type-B, respectively (as shown in Figure 6b,e). The surface atomic arrangements of all crystal planes in Regions C1 and C2 can be represented by atomic group configuration Type-C (as shown in Figure 6a). Likewise, the surface atomic arrangements of all crystal planes in Region D are primarily composed of atomic group configuration Type-D. The surface atomic arrangements of crystal planes in the Regions E1 and E2 regions are represented by a mixture of Type-D and Type-A or Type-B atomic groups (as shown in Figure 6c).

Given the analysis of the etching mechanism in Section 2.2, it can be inferred that the etching process on crystal planes can be seen as repetitively removing specific types of atomic groups. Consequently, if a basic crystal plane can be identified to measure the detachment rate of the type of atomic groups, the etching rates of various crystal planes can be calculated using geometric parameter differences from planes with identical surface atomic configurations. The etching rates of crystal planes with the same surface atomic configuration can be obtained by this method. As shown in Figure 5b, the crystal planes within the Regions A and B can each be represented by atomic groups on R$\left(0\;1\;\bar{1}\;1\right)$ and r$\left(0\;\bar{1}\;1\;1\right)$ planes (referred to as Type-A and Type-B, respectively). Therefore, they can serve as the basic crystal planes for this region. Utilizing Equation (1), the detachment rates of Type-A and Type-B atomic groups can be solved, and based on geometric parameter information among different crystal planes within this region, equations for the etching rates of other crystal planes within Regions A and B can be constructed. When the crystallographic direction Y$\langle 0\;1\;\bar{1}\;0\rangle$ rotates clockwise by 5° around X$\langle 2\;\bar{1}\;\bar{1}\;0\rangle$, all crystal planes within Regions E1 and E2 are captured. Crystal planes composed of Type-D atomic groups, initially appearing in a mixture with Type-A or Type-D, serve as the basic crystal planes within this region, namely, $\left(0\;5\;\bar{5}\;11\right)$ and $\left(0\;\bar{2}\;2\;7\right)$ planes. It is worth noting that the arrangement of atomic configurations in quartz crystal results in the m$\left(0\;1\;\bar{1}\;0\right)$ and (0001) crystal planes having more regular surface atomic structures compared to other planes. Microscopically, completing one cycle involves etching two or three layers of surface atoms, which is not a reasonable method for determining etching depth. This is mainly because the (0001) plane, although typically representing the fastest etching direction [10], only etches three layers of surface atoms. Conversely, the etching rate of the R crystal plane is significantly slower than that of the (0001) plane, necessitating the etching of 42 layers of surface atoms to complete one cycle. This characteristic goes against basic physical intuition. Therefore, although the m$\left(0\;1\;\bar{1}\;0\right)$ and (0001) planes can serve as basic crystal planes for analyzing atomic groups Type-C and Type-D, they do not effectively reflect microscopic geometric etching depth information. For this reason, neighboring crystal planes, $\left(0\;9\;\bar{9}\;1\right)$ and $\left(0\;1\;\bar{1}\;16\right)$, respectively, were selected as the basic crystal planes for Regions C1 and D, thus obtaining etching rate information for Type-C1 and Type-D. A similar approach was employed in selecting the $\left(0\;\bar{13}\;13\;6\right)$ plane as the basic crystal plane for Region C2.

## 3. Result and Discussion

### 3.1. Experiment

**Figure 7:**
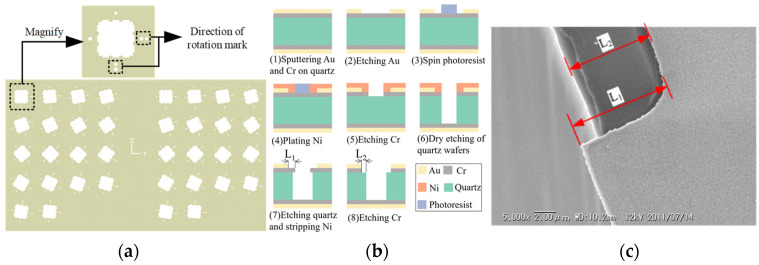
Experimental scheme for obtaining etching rate of quartz crystal plane. (**a**) Mask design scheme; (**b**) etching process; (**c**) SEM image of the wafer after etching.

To obtain etch rate data for quartz crystal planes, a 2 mm square mask with rotational direction markers was designed on the surface of X-cut quartz wafers, as shown in Figure 7a. This mask design allows for obtaining etch rate information for four crystal planes simultaneously, providing convenience for subsequent measurements. Rotating the square mask counterclockwise in 5° increments provides additional etching rate data for crystal orientations within 360°. The specific experimental process for obtaining the etch rate of crystal planes is depicted in Figure 7b. In this study, the thicknesses of the Au and Cr sputtered masks were 60 nm and 200 nm, respectively. Deep reactive ion etching (DRIE) technology was utilized to etch through the quartz wafer, resulting in well-defined vertical sidewalls. For the wet etching process, a saturated NH_4_HF_2_ solution was employed as the etchant for the quartz wafer with a thickness of 100 μm, which was immersed in an 80 ± 2 °C water bath for one hour. Notably, no stirring apparatus was utilized in the experimental procedure of this study. Figure 7c shows details captured by a scanning electron microscope (SEM) after etching the quartz wafer. The etch rate of the crystal faces can be calculated by measuring and recording the L_1_ and L_2_ positions in Figure 7b (V = (L_1_ − L_2_)/T, where T is the etching time). To reduce measurement errors, the etched morphologies of the two sets of identical masks in Figure 7a were measured individually, and the average value was used as the final etch rate data for each crystal plane.

### 3.2. Result Analysis and Discussion

Building upon the analysis presented in the Section 2, the relationship between basic crystal planes and macroscopic etch rates was established to determine micro-etch rates for each type of atomic group configuration. By partitioning surface atoms on different crystal planes and gathering geometric parameter information at a microscopic scale, micro-etch rates for each crystal plane could be determined. For instance, by measuring the geometric parameters of the basic crystal planes R, r, $\left(0\;9\;\bar{9}\;1\right)$, and $\left(0\;1\;\bar{1}\;16\right)$, the mapping relationship between macroscopic etch rates and microscopic atomic groups, as per Equation (1), was constructed to derive micro-detachment rates of atomic groups, as shown in Table 1.

**Figure 8:**
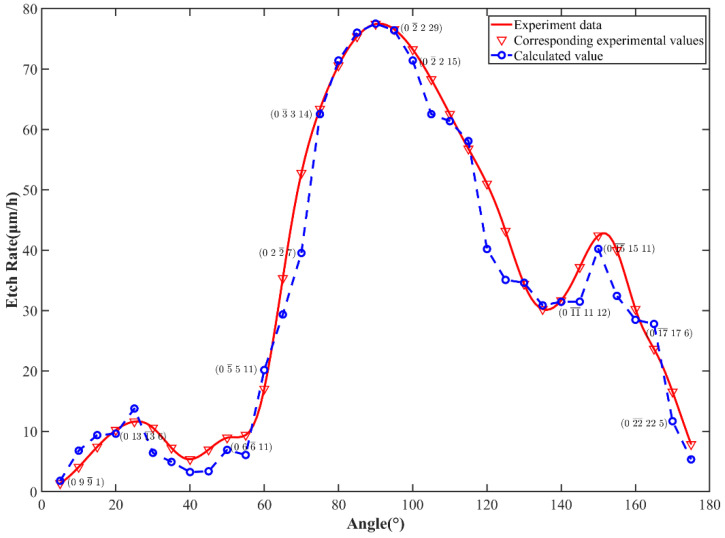
Comparison between experimental data and calculated results.

Table 1 reveals that the detachment rate of Type-D atomic groups significantly exceeds that of other cluster configurations. This is related to the fact that the D region evolves from the surface atomic group configuration of the (0001) crystal plane, which theoretically has a higher atomic group detachment rate. This indicates that the method of atomic group classification based on bond angles has a certain feasibility. Figure 8 compares the model-calculated etch rates with the experimental etch rates. The overall trends of increase or decrease in computed etch rates mirror those of experimental etch rates to a certain extent, suggesting the viability of the proposed micro-etch rate solving model. This indirectly indicates that the method of classifying the atomic group based on surface atomic bond angle characteristics can reasonably distinguish different crystal planes. By calculating the mean absolute error (MAE) and root mean square error (RMSE) between all experimental values and calculated values, the results were only 3.21 and 4.45, respectively. Table 2 provides specific etch rate information for certain crystal planes and the model calculation results. The table shows that the minimum absolute error was 1.36, while the maximum absolute error was 10.76, indicating a significant deviation from the experimental data. Consequently, Figure 8 illustrates a notable discrepancy between the calculated and experimental results for some crystal faces, such as the $\left(0\;2\;\bar{2}\;7\right)$ plane. This discrepancy arises because the proposed calculation model is relatively simplistic, treating the atomic group detachment process as a whole and not fully accounting for the impact of the number of surface atomic groups or sub-cluster configurations on the etching model. Furthermore, during the partitioning of surface atomic groups, certain individual dangling atomic structures may be overlooked (e.g., as depicted in Figure 6b), leading to computational inaccuracies to some extent due to this simplification method.

This study chose multiple basic crystal planes as the foundation for etching analysis. It segmented the surface atoms of diverse crystal planes into atomic groups and developed a model to delineate etching rate relationships among basic crystal planes and their adjacent counterparts. Preliminary research suggests the viability of this approach. A review of the literature on etch rate models revealed that the current classic step-flow model is based on specific reference crystal planes to construct etch rate equations at both macro and micro scales. This model has been applied to silicon [21], simplified sapphire [22], and gallium nitride atomic models [23]. While these models have produced satisfactory computational results, they involve intricate processes of step and terrace structure partitioning and solving. However, the helical structural features in the atomic arrangement of quartz crystals pose challenges in identifying step structures during the partitioning process of certain crystal planes, thus limiting its applicability to quartz materials. This paper simplified the analysis of complex crystal planes using a bond angle-based atomic group division method and developed an etch rate model based on this approach. Although the alignment between the calculated and experimental results needs further improvement, the proposed method offers a new perspective for analyzing and dividing crystal planes in complex materials. Additionally, the etch rate model in this paper relies solely on microscopic geometric information between different crystal planes for analysis, without fully considering the influence of neighboring atomic positions and quantities on the etching process. This limitation affects the accuracy of the proposed etch model to some extent but also provides an opportunity for further optimization. This consideration parallels the approach in reference [29], which associates etch rates with atomic configurations based on specific surface atomic neighbors and solves the detachment rates of atomic groups by simultaneously setting up equations between crystal planes. However, this analytical method evidently demands analyzing additional crystal planes (beyond those within the X-cut shape) and introducing more unknown variables. Due to the lack of complete 3D experimental etch rate data for quartz crystals, this study refrained from further research and expansion. However, this does not imply limitations in the method’s application. When the distance between adjacent crystal planes is sufficiently small, there must be a significant similarity in the arrangement of surface atoms. This similarity is primarily determined by the microscale atomic structure of the crystal. Thus, partitioning the number and spatial neighbor positions of atomic groups on different crystal planes will provide more valuable physical information at microscopic level. This will further enhance the computational accuracy of the proposed model, but also entail the need for more time and effort to analyze diverse crystal planes. Additionally, it will require more comprehensive support from macroscopic etch rate data.

## 4. Conclusions

A simplified model of the quartz crystal lattice was developed by simplifying the arrangement of oxygen atoms within the quartz unit cell. Utilizing the bond angle characteristics of this model, an analysis of the etching process was conducted on several representative quartz crystal planes. It was then approximated that the etching process of the crystal involves cyclic detachment of atomic groups based on the bond angle characteristics, thus establishing an etching rate model based on the geometric parameters of crystal planes. Furthermore, the proposed method was extended to include crystal planes within the X_cut of quartz, and the etching rates of faces within the cut were determined based on the etching rate model. Experimental results demonstrated good agreement between the calculated results of the proposed model and experimental data, indicating the feasibility and validity of the approach. This approach of categorizing crystal planes based on angular features introduces a novel perspective for investigating the anisotropy of crystal plane etch rates in complex crystal materials, offering theoretical backing for optimizing MEMS etching processes.

## Figures and Tables

**Table 1:** Group detachment rates of some basic crystal planes.

Basic Crystal Plane	$\left(0\;1\;\bar{1}\;1\right)$	$\left(0\;\bar{1}\;1\;1\right)$	$\left(0\;9\;\bar{9}\;1\right)$	$\left(0\;1\;\bar{1}\;16\right)$
Group type	Type-A	Type-B	Type-C	Type-D
Depth information (Å)	152.679	152.679	54.3125	140.847
Layer information	42	42	54.3125/4.913	140.847/5.404
Corresponding position angle (°)	89.984	90.015	89.942	89.932
Experimental data (μm/h)	5.4	34.6	1.7	76
Detachment rate	0.00084	0.0053	0.003	0.0207

**Table 2:** Error between calculation and experimental results of some crystal planes.

Crystal Plane	Experimental Data (μm/h)	Model Calculation Result (μm/h)	Absolute Error
(0 17 −17 6)	8	9.36	1.36
(0 10 −10 9)	7.2	4.91	2.29
(0 4 −4 11)	35.3	29.33	5.97
(0 −2 2 15)	73.2	71.39	1.81
(0 −5 5 11)	50.9	40.17	10.76
(0 −10 10 9)	37.1	31.45	5.56

## Data Availability

Data are contained within the article.
